# Copy number variation of *Ppd-B1* is the major determinant of heading time in durum wheat

**DOI:** 10.1186/s12863-019-0768-2

**Published:** 2019-07-29

**Authors:** Tobias Würschum, Matthias Rapp, Thomas Miedaner, C. Friedrich H. Longin, Willmar L. Leiser

**Affiliations:** State Plant Breeding Institute, University of Hohenheim, 70593 Stuttgart, Germany

**Keywords:** Durum wheat, Heading time, *Ppd-B1*, Copy number variation, CNV

## Abstract

**Background:**

Heading time is an important adaptive trait in durum wheat. In hexaploid wheat, *Photoperiod-1* (*Ppd*) loci are essential regulators of heading time, with *Ppd-B1* conferring photoperiod insensitivity through copy number variations (CNV). In tetraploid wheat, the D-genome *Ppd-D1* locus is absent and generally, our knowledge on the genetic architecture underlying heading time lacks behind that of bread wheat.

**Results:**

In this study, we employed a panel of 328 diverse European durum genotypes that were evaluated for heading time at five environments. Genome-wide association mapping identified six putative QTL, with a major QTL on chromosome 2B explaining 26.2% of the genotypic variance. This QTL was shown to correspond to copy number variation at *Ppd-B1*, for which two copy number variants appear to be present. The higher copy number confers earlier heading and was more frequent in the heat and drought prone countries of lower latitude. In addition, two other QTL, corresponding to *Vrn-B3* (*TaFT*) and *Ppd-A1*, were found to explain 9.5 and 5.3% of the genotypic variance, respectively.

**Conclusions:**

Our results revealed the yet unknown role of copy number variation of *Ppd-B1* as the major source underlying the variation in heading time in European durum wheat. The observed geographic patterns underline the adaptive value of this polymorphism and suggest that it is already used in durum breeding to tailor cultivars to specific target environments. In a broader context our findings provide further support for a more widespread role of copy number variation in mediating abiotic and biotic stress tolerance in plants.

**Electronic supplementary material:**

The online version of this article (10.1186/s12863-019-0768-2) contains supplementary material, which is available to authorized users.

## Background

Durum wheat (*T. durum*) is the main source for pasta and semolina products. Heading time is an important trait in durum wheat breeding, as it is a key element of adaptation and thus also affects yield. Durum is traditionally grown in the Mediterranean countries, i.e. in Southern Europe, West Asia and North Africa, due to the climatic conditions with mild winters, spring rain and dry summers. However, in the last decades durum cultivation has made its way northwards to higher latitudes, for example to Germany [1]. Generally, spring and winter types can be distinguished, with the winter hardiness required for cultivation in Central Europe originating from eastern, continental countries, including Russia, the Ukraine, and some other countries surrounding the Black Sea [2, 3]. This distinction is, however, somewhat arbitrary, as in the Mediterranean the spring type is often sown in autumn to take advantage of the humidity during winter and spring, and to escape heat and drought stress in summer by an early ripening. The latter is facilitated by an early heading, which requires some degree of photoperiod insensitivity.

In wheat, heading time is controlled by the vernalization (*Vrn*), photoperiod (*Ppd*), and earliness per se (*Eps*) pathways [4, 5]. Wheat requires a certain day-length to promote heading, but photoperiod-insensitive alleles at the *Ppd* loci can induce heading irrespective of day length. In hexaploid wheat, *Ppd-D1* has long been recognized as an important locus for flowering time and shown to encode a pseudo-response regulator [6, 7]. The *Ppd-1* loci form a homoeoallelic series on the short arms of group 2 chromosomes with *Ppd-D1* having the largest influence on heading time in bread wheat [8]. Additionally, photoperiod insensitivity has been shown to co-segregate with *Ppd-B1*, but no likely causal mutation could be identified [9]. Instead, *Ppd-B1* was shown to be present in different copy numbers, with higher copy number variants conferring photoperiod insensitivity and resulting in earlier heading [8, 10–13]. Also tetraploid wheat species show a wide variation in heading time and photoperiod insensitivity is well known, but due to the absence of the D genome, the observable variation must be independent from *Ppd-D1* [14]. In general, our understanding of the genetic control underlying heading time in durum wheat lacks behind that of hexaploid bread wheat.

The aim of this study was to bridge this gap and to evaluate the possible contribution of copy number variation to the adaptation of heading time in durum wheat. To this end, we employed a large panel of 328 diverse elite durum genotypes originating from across Europe to analyze variation in heading time and dissect the underlying genetic architecture.

## Results

Heading time was recorded in the panel of 328 diverse European durum lines grown at five environments, which revealed a range of 20 days between the earliest and the latest heading genotypes (Additional file 1: Table S2, Additional file 1: Figure S1). The genotypic variance (${\sigma}_G^2$) as well as the genotype-by-environment interaction variance (${\sigma}_{G\times E}^2$) were both significantly different from zero (*P* < 0.001) and the ratio between them was 9.1, yielding a high heritability of 0.88.

**Fig. 1:**
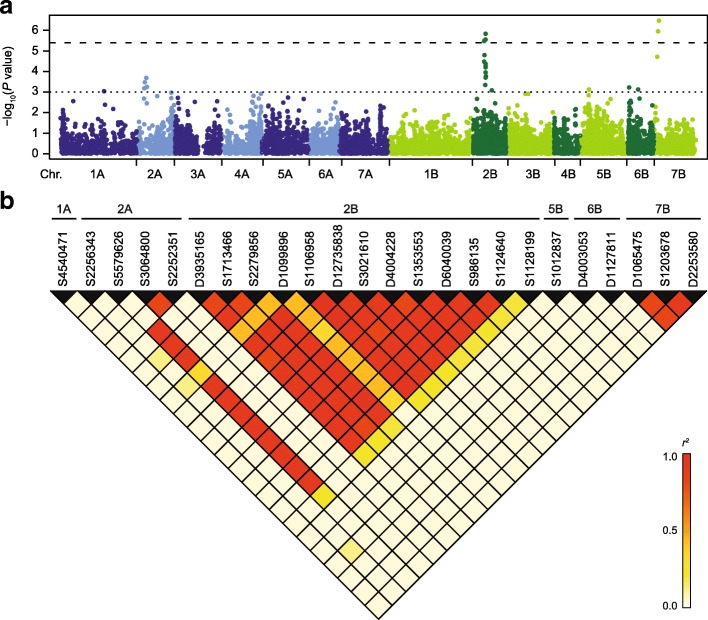
Identification of heading time QTL in durum wheat. **a** Manhattan plot showing results from the genome-wide scan for marker-trait associations for heading time. The dashed line indicates the significance threshold (Bonferroni-corrected *P* < 0.01) and the dotted line the exploratory threshold (*P* < 0.001). **b** Linkage disequilibrium between the significantly associated markers

Genome-wide association mapping revealed 24 marker-trait associations at the exploratory significance threshold (*P* < 0.001), of which some of the associations on chromosomes 2B and 7B reached the Bonferroni-corrected significance threshold (Table 1, Fig. 1a). Thirteen marker-trait associations were identified on chromosome 2B, most of them at around 40.7 cM. Analysis of the linkage disequilibrium (LD) between the significantly associated markers revealed a high LD between the markers on 2B, as well as one of the markers genetically mapped to chromosome 2A, indicating that the latter may actually be located on chromosome 2B (Fig. 1b). Otherwise, there was low LD between the putative QTL. Correcting for collinearity by a joint analysis of the significantly associated markers supported the conclusion that they likely correspond to six putative QTL located on chromosomes 1A, 2A, 2B, 5B, 6B and 7B (Table 1). Together, these putative QTL explained 55.06% of the genotypic variance. The highest proportion of genotypic variance was attributable to the putative QTL on chromosome 2B, explaining 26.15%. Even when considering this QTL in the linear model, the putative QTL on chromosome 7B still explained 9.47% of the genotypic variance and the QTL on 2A explained 5.32%.

**Table 1:** Marker-trait associations for heading time identified in the durum wheat panel

Marker	Chr.	Pos. (cM)^a^	Pos. (Mbp)^b^	*P* value	*p*_*G-single*_^d^	*p*_*G-joint*_^e^	*p*_*G-Ppd*_^f^	Effect	Freq.^g^(SD, WD)
S4540471	1A	145.07	526,685,715	9.02e-4	5.88	1.56	1.43	0.76	0.60 (0.66, 0.57)
S2256343	2A	23.92	36,364,298	6.51e-4	20.78	5.32	5.22	−1.35	0.45 (0.73, 0.28)
S5579626	2A	25.56	35,596,685^c^	3.32e-4	20.81	0.85	0.87	−1.80	0.15 (0.25, 0.10)
S3064800	2A	31.13	35,627,791	2.04e-4	4.75	1.42	1.48	−0.83	0.20 (0.25, 0.18)
S2252351	2A	33.58	35,846,102	5.66e-4	4.33	0.05	0.05	−0.84	0.20 (0.23, 0.18)
*Ppd-B1* CNV	2B				22.64		22.92	−2.00	
D3935165	2B	36.35	53,704,532	3.27e-6	23.05	0.01	0.00	−1.86	0.15 (0.24, 0.10)
S1713466	2B	36.35	53,972,352	3.31e-5	24.24	0.40	0.39	−1.92	0.17 (0.27, 0.11)
S2279856	2B	37.15	56,191,088	1.62e-5	21.72	0.28	0.36	−1.85	0.15 (0.25, 0.09)
D1099896	2B	39.51	53,406,376	4.45e-4	18.18	0.44	0.43	−1.89	0.11 (0.28, 0.01)
S1106958	2B	40.74	53,701,140	1.45e-6	24.84	26.15	3.72	−1.98	0.15 (0.26, 0.09)
D12735838	2B	40.74	53,067,983	2.81e-6	25.04	0.24	0.22	−1.85	0.17 (0.29, 0.10)
S3021610	2B	40.74	53,972,355	4.24e-5	23.31	0.00	0.00	−1.92	0.16 (0.26, 0.10)
D4004228	2B	40.74	56,011,661	6.34e-5	22.76	0.08	0.06	−1.81	0.16 (0.26, 0.10)
S1353553	2B	40.74	54,098,441	1.13e-4	21.42	0.05	0.05	−1.84	0.16 (0.24, 0.11)
D6040039	2B	40.74	53,972,355	1.98e-4	21.59	0.35	0.30	−1.73	0.16 (0.26, 0.11)
S986135	2B	40.99	54,516,891	5.42e-5	22.86	0.05	0.10	−1.91	0.15 (0.26, 0.09)
S1124640	2B	41.86	54,468,610	1.67e-4	20.99	0.13	0.10	−1.87	0.15 (0.24, 0.10)
S1128199	2B	61.42	–	8.21e-4	15.75	1.02	0.92	−1.63	0.15 (0.18, 0.13)
S1012837	5B	29.33	82,403,619	7.51e-4	10.66	3.93	4.19	−1.05	0.30 (0.48, 0.19)
D4003053	6B	5.12	49,777,628	6.04e-4	4.39	3.57	3.70	0.68	0.74 (0.74, 0.74)
D1127811	6B	34.67	620,269,666	7.54e-4	18.77	3.91	3.79	−1.42	0.23 (0.44, 0.11)
D1065475	7B	11.81	7,619,225	1.94e-5	7.22	0.01	0.00	0.76	0.57 (0.73, 0.46)
S1203678	7B	14.14	9,823,914	1.11e-6	8.21	0.07	0.16	−0.87	0.48 (0.68, 0.40)
D2253580	7B	16.94	9,998,428	3.36e-7	9.15	9.47	9.34	0.85	0.54 (0.70, 0.44)

^a^Genetic map positions as provided by Diversity Arrays Technology

^b^Physical position on the durum wheat reference genome Maccaferri et al. [35]

^c^Physical position on chromosome 2A (E value 1e-17), alternative position on 2B at 53,691,676 (E value 3e-20)

^d^Proportion of explained genotypic variance of the marker singly

^e^Proportion of explained genotypic variance in a joint fit of all markers in the order of the strength of their association

^f^Proportion of explained genotypic variance in a joint fit of all markers but with *Ppd-B1* CNV included and modelled first

^g^Frequency of the earliness-conferring allele; in brackets the frequency in the spring type (SD) and winter type (WD) durum lines is shown

**Fig. 2:**
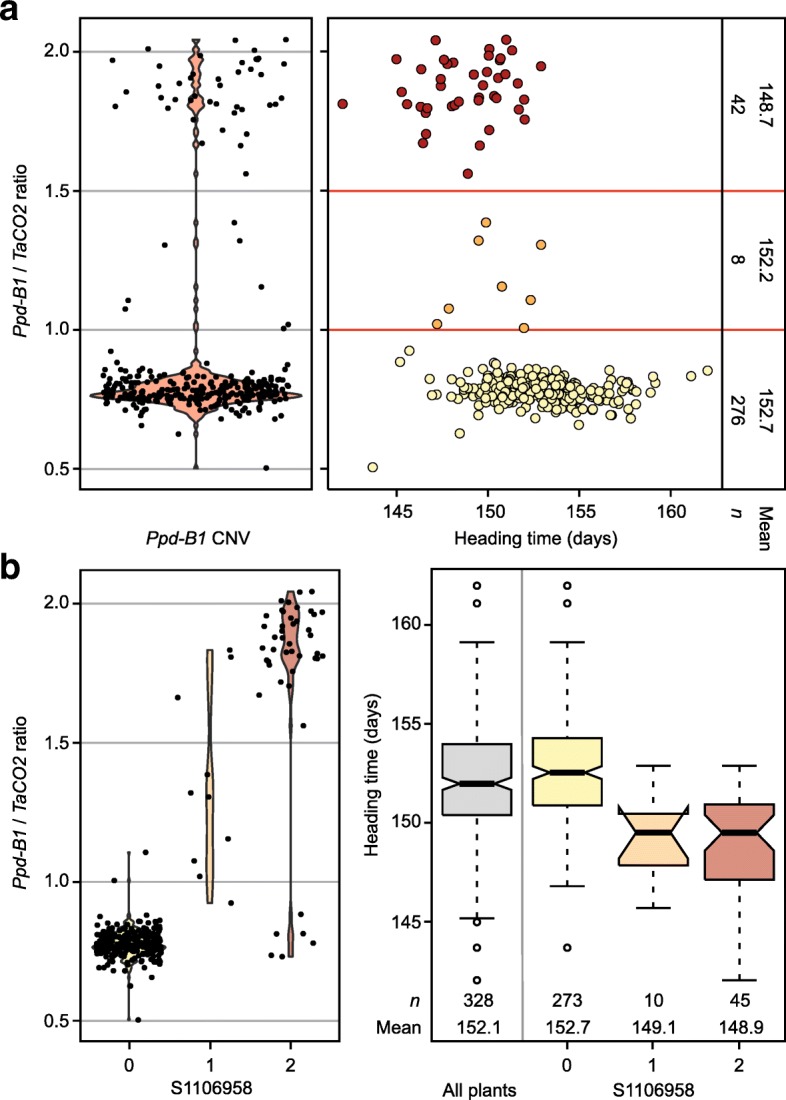
Copy number variation at *Ppd-B1* and its effect on heading time. **a** *Ppd-B1* copy number variation. The red horizontal lines indicate arbitrarily defined copy number classes based on the distribution of the *Ppd-B1* / *TaCO2* (internal positive control) ratio. **b** *Ppd-B1* copy number for the alleles at the marker explaining the highest proportion of genotypic variance

We assessed copy number variation of *Ppd-B1* as the ratio between *Ppd-B1* and the internal control *TaCO2*. This revealed two major groups of signal ratios, one with 276 genotypes between 0.5 and 1.0, mainly at around 0.75, and another with 42 genotypes between 1.5 and 2.1 (Fig. 2a). Eight genotypes had a signal ratio between these two. The group with the higher *Ppd-B1* / *TaCO2* signal ratio headed on average 4 days earlier (*P* < 0.01) than the group of genotypes with the lower ratio. The correlation between the significantly associated markers on chromosome 2B and *Ppd-B1* copy number variation was high, indicating that they identify the *Ppd-B1* locus (Additional file 1: Table S3). This was substantiated by the finding that *Ppd-B1* copy number variation captures the genotypic variation of the 2B QTL when included in the linear model (Table 1). Accordingly, the two alleles of the most strongly associated marker on 2B, S1106958, separated the two *Ppd-B1* / *TaCO2* signal ratio groups well (Fig. 2b). Interestingly, most of the genotypes with a signal ratio between the two major groups were scored as being heterozygous for this marker. This is unlikely to be a technical artifact as other significantly associated SNP markers of the 2B QTL supported the heterozygous scoring.

**Fig. 3:**
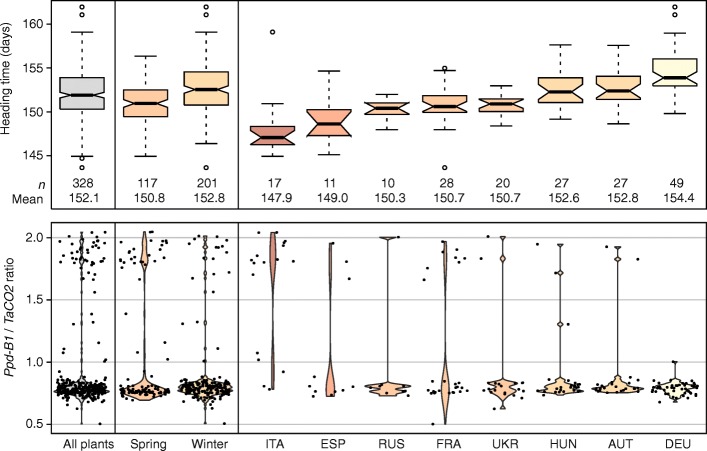
Heading time and copy number variation of *Ppd-B1* in genotypes from different countries of origin. ITA, Italy; ESP, Spain; RUS, Russian Federation; FRA, France; UKR, Ukraine; HUN, Hungary; AUT, Austria; DEU, Germany

We next evaluated heading time and *Ppd-B1* copy number variation dependent on the geographic origin of the durum genotypes. The earliest heading genotypes were those from Italy with a mean of 147.9 days, whereas the genotypes from Germany were on average the latest heading ones with 154.4 days (Fig. 3). Copy number variation of *Ppd-B1* followed this pattern, with many Italian genotypes having the high signal ratio, but also several of the French cultivars, presumably those grown in the southern, Mediterranean regions. By contrast, all of the substantially later heading German lines were of the lower signal ratio group.

## Discussion

Heading time is an important adaptive trait in small grain cereals, including durum wheat. We observed a significant genotypic variation and a large range of 20 days. This is in part attributable to the broad sampling of the diversity panel, with genotypes covering the durum growing countries in Europe from North to South (Fig. 3). Nevertheless, we also observed a substantial variation within each country of origin. Besides yield and agronomic traits, quality is a major target in durum wheat breeding. To introgress available variation, crosses are also made between cultivars from different geographic origin. This requires a subsequent fine-tuning of heading to the target region, which might be facilitated by a better understanding of the genetic control underlying heading time.

### Genetic architecture of heading time in durum wheat

Genome-wide association mapping identified six putative QTL that explained approximately half of the genotypic variance. The other half is likely attributable to small-effect QTL that cannot be detected or potentially also to epistasis [11, 13, 24]. Three of the identified putative QTL, located on chromosomes 1A, 5B and 6B, can be regarded as small-effect QTL, while the other three QTL were found to each explain more than 5% of the genotypic variance: the major QTL on chromosome 2B, as well as the QTL on 2A and 7B (Table 1). The QTL on chromosome 2A likely corresponds to *Ppd-A1*, as the physical position of the most strongly associated markers is close to this gene (Additional file 1: Figure S2). *Ppd-1* exists as a homoeologous series on group 2 chromosomes, but in hexaploid wheat no photoperiod insensitive allele has been described for the A genome homoeologue. For tetraploid wheat, by contrast, Wilhelm et al. [25] showed insensitivity to be caused by two deletions upstream of the *Ppd-A1* gene, that remove a region also deleted in the photoperiod insensitive *Ppd-D1a* allele. Bentley et al. [26] found neither of the two alleles in wild tetraploid wheat, but showed them to be widespread in modern durum wheat, suggesting that they originated after domestication and were subsequently selected for to improve adaptation. In European durum, the frequency of these alleles was reported to be highest in cultivars from the southern European countries Italy and Spain, in line with a much higher selection pressure for early heading in these more heat and drought prone countries. In our panel, the analysis of the allele frequencies of the most strongly associated marker with regards to the cultivars’ country of origin confirmed this trend (Additional file 1: Figure S3).

The QTL on chromosome 7B corresponds to *Vrn-B3*, as again the physical position of the most strongly associated markers coincided with the location of the gene (Additional file 1: Figure S2). *Vrn-B3* encodes the cereal orthologue of the Arabidopsis *FLOWERING LOCUS T* (*FT*), that in wheat is induced in response to long days to promote flowering, thus mediating day-length response [4, 5, 27]. Taken together, the genetic architecture of heading time in durum wheat is complex, controlled by a few medium- to large-effect QTL and numerous small-effect QTL that jointly facilitate fine-tuning of heading time to a broad range of environmental conditions.

### *Ppd-B1* copy number variation shapes heading time in durum

The major QTL for heading time was identified on chromosome 2B and shown to correspond to copy number variation at *Ppd-B1* (Figs. 1 and 2). For hexaploid wheat, Díaz et al. [9] reported 1–4 haploid copy numbers of *Ppd-B1*. For durum wheat, we found two groups for the *Ppd-B1* / *TaCO2* signal ratio, suggesting two *Ppd-B1* copy number variants in this durum panel. The genotypes with a signal ratio between these two groups were scored as heterozygous by most of the SNP markers identifying this QTL, indicating these lines to be either heterozygous or more likely heterogenous for this locus. The Italian cultivar ‘Svevo’ used for the durum reference genome belonged to the higher signal ratio group, but *Ppd-B1* was not annotated on the B genome. Thus, the haploid copy number of the two groups, as well as allelic variants of the different copies need to be determined by further research on a molecular level.

As in hexaploid wheat, a higher copy number of *Ppd-B1* was found to lead to earlier heading. Under our field conditions, the difference between the two copy number variants was four days, which however, is likely dependent on the environment. Würschum et al. [12] have recently reported that in hexaploid wheat *Ppd-B1* copy number variation shows a geographical pattern following latitude, with a higher frequency of the photoperiod-insensitive high copy number variants in the countries of lower latitude. We observed a similar pattern for durum wheat (Fig. 3), as for example most Italian cultivars carried the higher copy number variant, while almost all varieties from Austria and Germany carried the lower copy number variant. This illustrates the adaptive value of *Ppd-B1* copy number variation in durum and shows that it is already actively utilized in durum wheat breeding.

## Conclusions

In this study, we evaluated a broad panel of durum genotypes and found a complex genetic architecture underlying the variation in heading time. *Ppd-A1* and *Vrn-B3* (*FT*) were found to account for a substantial proportion of the genotypic variance and both loci may thus be targets for a marker-assisted selection. Moreover, copy number variation of *Ppd-B1* on chromosome 2B was identified as having the largest impact on heading time and thus latitudinal adaptation in European durum wheat. Collectively, our results corroborate findings from hexaploid bread wheat on the importance of *Ppd-B1* copy number variation, and in a broader context may substantiate a more widespread role of copy number variation in mediating abiotic and biotic stress tolerance in plants (e.g. [9, 28–34]).

## Methods

### Plant material and experimental design

This study is based on a durum wheat diversity panel, comprising registered cultivars obtained for research purposes from the breeding companies Südwestsaat, Syngenta, Saatzucht Donau, RAGT, Florimond-Desprez, and Limagrain, as well as breeding lines from the breeding program of the State Plant Breeding Institute at the University of Hohenheim (Additional file 1: Table S1) [3, 15]. The authors checked and confirmed all plant material to be durum wheat. The breeding lines represent durum wheat breeding material that was only propagated for the purpose of this research and is available upon request. The lines included in this study are adapted to the Northern Mediterranean, as well as to Central and Eastern European climatic conditions. The material can be classified as spring or winter types, but can be sown in autumn as long as the temperature without snow coverage does not drop below − 10 °C. The panel was grown in a winter cropping system, i.e. was sown in October and the plants reached maturity in July of the following year. The field experiments were performed at three locations in 2016 and at two locations in 2017, in accordance with local legislation. The genotypes were grown in observation plots with two rows of 1 m length, arranged as a partially replicated design with a replication factor of 1.18 [16]. In the 2016 season, the locations were Hohenheim (HOH), Oberer Lindenhof (OLI) and Eckartsweier (EWE), while in the 2017 season the location Eckartsweier was omitted, resulting in a total of five environments. Heading time was recorded as the day in the year, when 75% of the ears of a plot had fully emerged from the flag leaf. Phenotypic analysis was done as described by Miedaner et al. [17]. In brief, best linear unbiased estimates (BLUEs) were estimated across environments, assuming fixed effects for the genotype in the following model: *y*_*ijk*_ *= μ + g*_*i*_ *+ e*_*j*_ *+ ge*_*ij*_ *+ b*_*jk*_ *+ ɛ*_*ijk*_, where *y*_*ijk*_ was the phenotypic observation of the *i*th durum genotype at the *j*th environment in the *k*th incomplete block, *μ* was an intercept term, *g*_*i*_ the genetic effect of the *i*th genotype, *e*_*j*_ the effect of the *j*th environment, *ge*_*ij*_ the genotype-by-environment interaction, *b*_*jk*_ the effect of the *k*th incomplete block at the *j*th environment, and *ɛ*_*ijk*_ was the residual. Variance components were estimated in a full random model based on a restricted maximum likelihood (REML) method and their significance tested by likelihood ratio tests. Heritability (*h*^*2*^) was estimated following the approach suggested by Piepho and Möhring [18]. All statistical analyses were performed using the statistical software R [19] and ASReml-R 3.0 [20].

### Genotypic and molecular analyses

The panel was genotyped by genotyping-by-sequencing at Diversity Arrays Technology (Yarraluma, Australia) [21]. The dominant silico-DArTs and the co-dominant single nucleotide polymorphism (SNP) markers are in the following denoted by their clone ID and the marker-type prefix ‘D’ or ‘S’, respectively. Markers showing more than 20% missing values or a minor allele frequency lower than 5% were removed from the initial marker set, resulting in 20,276 markers. The 4.85% missing values weres imputed by the software package LD-kNNi, with an imputation accuracy of 0.99 [22]. After the imputation, markers with a minor allele frequency lower than 5% were again removed, resulting in 12,550 markers with known map position (Wheat ConsensusMap Version 4). Genotyping of *Ppd-B1* copy number variation followed the protocol reported by Díaz et al. [9] with minor modifications [11]. *TaCO2* served as internal control and the ratio *Ppd-B1* / *TaCO2* was used to assess copy numbers of *Ppd-B1*.

Association mapping was done with the software package ‘GenABEL’ [23] with a linear mixed model incorporating a kinship matrix as described by Miedaner et al. [17]. A Bonferroni-corrected significance threshold of *P* < 0.05 and an exploratory threshold of *P* < 0.001 were used to identify significant marker-trait associations. The proportion of genetic variance explained by the putative QTL was estimated by fitting the significant markers in linear models, either singly or jointly in the order of the strength of their association, and dividing the resulting sums of squares values of each marker by the heritability of the trait.

## Additional file

Additional file 1: **Table S1.** Genotypes included in this study. **Table S2.** Summary statistics for heading time. **Table S3.** Correlations between the significantly associated markers on chromosome 2B and *Ppd-B1* copy number variation (ratio *Ppd-B1* / *TaCO2*) . **Figure S1.** Histogram of the heading time BLUEs. **Figure S2.** Results from the genome-wide scan for marker-trait association for heading time for chromosomes 2A, 2B and 7B, with the markers plotted according to their physical position in the wild emmer reference genome [36]. **Figure S3.** Allele frequency of marker S2256343, as a proxy for *Ppd-A1*, dependent on the cultivars’ country of origin. (DOCX 206 kb)

## Data Availability

The datasets supporting the conclusions of this article are included within the article and its additional files.

## Abbreviations

CNV: Copy number variation
DArT: Diversity Arrays Technology
EWE: Eckartsweier
HOH: Hohenheim
LD: Linkage disequilibrium
OLI: Oberer Lindenhof
*Ppd*: *Photoperiod* loci
QTL: Quantitative trait loci
SNP: Single nucleotide polymorphism
*Vrn*: *Vernalization* loci
