# Supplementary material for: Copy number variation of Ppd-B1 is the major determinant of heading time in durum wheat
Source: BMC Genet. 2019 Jul 29;20:64. doi: 10.1186/s12863-019-0768-2 (PMC6664704; doi:10.1186/s12863-019-0768-2)
Supplement: Supplementary file 1 — Table S1. Genotypes included in this study. Table S2. Summary statistics for heading time. Table S3. Correlations between the significantly associated markers on chromosome 2B and Ppd-B1 copy number variation (ratio Ppd-B1 / TaCO2) . Figure S1. Histogram of the heading time BLUEs. Figure S2. Results from the genome-wide scan for marker-trait association for heading time for chromosomes 2A, 2B and 7B, with the markers plotted according to their physical position in the wild emmer reference genome [36]. Figure S3. Allele frequency of marker S2256343, as a proxy for Ppd-A1, dependent on the cultivars’ country of origin. (DOCX 206 kb) [file 12863_2019_768_MOESM1_ESM.docx]

**Copy number variation of *Ppd-B1* is the major determinant of heading time in durum wheat**

Tobias Würschum^🖂^, Matthias Rapp, Thomas Miedaner, C. Friedrich H. Longin and Willmar L. Leiser

State Plant Breeding Institute, University of Hohenheim, 70593 Stuttgart, Germany

🖂 Tobias Würschum, email: [tobias.wuerschum@uni-hohenheim.de](mailto:tobias.wuerschum@uni-hohenheim.de)

**Additional files**

**Additional file 1: Table S1.** List of the genotypes included in this study.

| Genotyp | Type^1^ | Status | Origin | Year of Admission | Heading | CNV TargetRef |
| --- | --- | --- | --- | --- | --- | --- |
| 05ETTRA4IN11 | s | Modern breeding line | FRA | NA | 154.99 | 0.75 |
| 12-HPW-10 | w | Modern breeding line | DEU | NA | 149.98 | 0.79 |
| 12-HPW-16 | w | Modern breeding line | DEU | NA | 153.67 | 0.79 |
| 2.002/08/01 | s | Modern breeding line | NA | NA | 149.83 | 1.39 |
| 2.003/07/02 | s | Modern breeding line | NA | NA | 151.77 | 0.78 |
| 2.005/09/01 | s | Modern breeding line | NA | NA | 149.13 | 0.76 |
| 2.016/09/01 | s | Modern breeding line | NA | NA | 154.61 | 0.74 |
| 2.016/09/02 | s | Modern breeding line | NA | NA | 154.73 | 0.75 |
| 2.016/09/03 | s | Modern breeding line | NA | NA | 150.84 | 0.77 |
| 2.018/05/01 | s | Modern breeding line | NA | NA | 151.05 | 0.81 |
| 2.019/07/09 | s | Modern breeding line | NA | NA | 152.20 | 0.76 |
| 2.025/09/01 | s | Modern breeding line | NA | NA | 149.57 | 0.78 |
| 2.027/05/04 | s | Modern breeding line | NA | NA | 153.63 | 0.73 |
| 2.028/09/01 | s | Modern breeding line | NA | NA | 147.36 | 1.90 |
| 2.034/09/01 | s | Modern breeding line | NA | NA | 151.07 | 0.74 |
| 2.037/09/01 | s | Modern breeding line | NA | NA | 151.61 | 0.78 |
| 2.040/09/02 | s | Modern breeding line | NA | NA | 156.36 | 0.78 |
| 2.043/07/02 | s | Modern breeding line | NA | NA | 149.53 | 0.79 |
| 2.074/08/02 | s | Modern breeding line | NA | NA | 151.50 | 0.81 |
| 2.075/08/01 | s | Modern breeding line | NA | NA | 152.25 | 0.76 |
| 2.093/07/02 | s | Modern breeding line | NA | NA | 151.24 | 0.75 |
| 6.001/99/01 | w | Old breeding line | DEU | NA | 157.26 | 0.81 |
| 6.002/08/401 | w | Modern breeding line | DEU | NA | 150.73 | 0.76 |
| 6.007/07/04 | w | Modern breeding line | DEU | NA | 157.62 | 0.71 |
| 6.008/03/01 | w | Modern breeding line | DEU | NA | 152.58 | 0.86 |
| 6.008/04/01 | w | Modern breeding line | DEU | NA | 158.97 | 0.83 |
| 6.008/04/03 | w | Modern breeding line | DEU | NA | 156.68 | 0.82 |
| 6.009/03/03 | w | Modern breeding line | DEU | NA | 153.19 | 0.82 |
| 6.011/97/04 | w | Modern breeding line | DEU | NA | 151.91 | 1.01 |
| 6.015/07/01 | w | Modern breeding line | DEU | NA | 152.19 | 0.75 |
| 6.018/99/02 | w | Modern breeding line | DEU | NA | 161.96 | 0.85 |
| 6.020/05/01 | w | Modern breeding line | DEU | NA | 157.76 | 0.68 |
| 6.021/06/05 | w | Modern breeding line | DEU | NA | 158.06 | 0.78 |
| 6.022/07/01 | w | Modern breeding line | NA | NA | 156.71 | 0.69 |
| 6.024/08/431 |  | Modern breeding line | DEU | NA | 155.57 | 0.78 |
| 6.024/08/431 | w | Modern breeding line | DEU | NA | 153.64 | 0.80 |
| 6.025/05/02 | w | Modern breeding line | DEU | NA | 153.64 | 0.81 |
| 6.026/05/03 | w | Modern breeding line | DEU | NA | 151.96 | 0.79 |
| 6.027/05/02 | w | Modern breeding line | DEU | NA | 153.92 | 0.80 |
| 6.029/04/02 | w | Modern breeding line | DEU | NA | 150.54 | 0.78 |
| 6.029/05/02 | w | Modern breeding line | DEU | NA | 152.97 | 0.80 |
| 6.029/07/01 | w | Modern breeding line | DEU | NA | 155.13 | 0.71 |
| 6.032/06/01 | w | Modern breeding line | DEU | NA | 151.05 | 0.81 |
| 6.034/04/03 | w | Modern breeding line | DEU | NA | 161.06 | 0.83 |
| 6.034/08/444 | w | Modern breeding line | DEU | NA | 156.04 | 0.78 |
| 6.035/02/01 | w | Modern breeding line | DEU | NA | 152.73 | 0.79 |
| 6.040/04/01 | w | Modern breeding line | DEU | NA | 153.46 | 0.82 |
| 6.040/05/01 | w | Modern breeding line | DEU | NA | 152.75 | 0.77 |
| 6.040/08/453 | w | Modern breeding line | DEU | NA | 153.28 | 0.77 |
| 6.044/08/456 | w | Modern breeding line | DEU | NA | 155.96 | 0.75 |
| 6.046/04/01 | w | Modern breeding line | DEU | NA | 154.56 | 0.80 |
| 6.047/02/02 | w | Modern breeding line | DEU | NA | 154.75 | 0.79 |
| 6.047/04/01 | w | Modern breeding line | DEU | NA | 153.88 | 0.83 |
| 6.047/07/01 | w | Modern breeding line | DEU | NA | 157.09 | 0.74 |
| 6.048/07/04 | w | Modern breeding line | DEU | NA | 158.04 | 0.73 |
| 6.048/08/457 | w | Modern breeding line | DEU | NA | 157.53 | 0.81 |
| 6.049/01/01 | w | Modern breeding line | DEU | NA | 156.58 | 0.85 |
| 6.051/04/04 | w | Modern breeding line | DEU | NA | 154.61 | 0.77 |
| 6.052/04/01 | w | Modern breeding line | DEU | NA | 154.06 | 0.81 |
| 6.052/04/05 | w | Modern breeding line | DEU | NA | 153.11 | 0.81 |
| 6.054/08/466 | w | Modern breeding line | DEU | NA | 153.65 | 0.80 |
| 6.065/07/02 | w | Modern breeding line | DEU | NA | 149.81 | 0.75 |
| 6.070/07/01 | w | Modern breeding line | DEU | NA | 153.49 | 0.76 |
| 6.073/06/01 | w | Modern breeding line | DEU | NA | 152.57 | 0.79 |
| 6.136/00/03 | w | Modern breeding line | DEU | NA | 154.09 | 0.80 |
| 6.155/00/02 | w | Modern breeding line | DEU | NA | 153.21 | 0.72 |
| ACNavigator | w | Old variety | CAN | 1999 | 146.75 | 0.82 |
| ADAMELLO | s | Old variety | ITA | 1985 | 148.04 | 1.96 |
| Agathe | w | Old variety | FRA | 1970 | 153.03 | 0.85 |
| Ajsberg1UKR8 | w | Old variety | UKR | NA | 152.35 | 0.77 |
| Ajsbergodesskij | w | Old variety | UKR | 1990 | 148.44 | 0.81 |
| Aksinit | w | Modern variety | RUS | 2004 | 151.26 | 2.01 |
| Alena | w | Modern variety | RUS | 2000 | 151.02 | 0.77 |
| ALEXIS | s | Modern variety | FRA | 2010 | 150.43 | 0.76 |
| AliyParus | w | Old variety | UKR | 1993 | 150.53 | 0.83 |
| Alpidur | w | Old variety | FRA | 1996 | 148.85 | 0.85 |
| Altar84 | w | Old variety | MEX | 1984 | 150.46 | 0.84 |
| Amazonka | w | Modern variety | RUS | 2009 | 149.71 | NA |
| AMILCAR | s | Modern variety | ESP | 2002 | 148.13 | 1.81 |
| Ankara98 | w | Old variety | TUR | 1998 | 150.37 | 0.88 |
| Anvergur | s | Modern variety | FRA | 2012 | 151.12 | 0.78 |
| Argonavt | w | Modern variety | UKR | 2002 | 151.27 | 0.83 |
| Arhipelag | w | Old variety | UKR | NA | 149.77 | 0.79 |
| Auradur | w | Modern variety | AUT | 2004 | 152.44 | 0.81 |
| BABYLONE | s | Modern variety | FRA | 2009 | 154.71 | 0.76 |
| Belfugitto | w | Old variety | ITA | 1973 | 159.10 | 0.81 |
| Berwidur | w | Old variety | DEU | 1993 | 154.11 | 0.83 |
| Biensur | s | Modern variety | FRA | 2001 | 150.59 | 1.97 |
| Burshtin | w | Modern variety | UKR | 2007 | 152.97 | 0.73 |
| Cakmak | w | Old variety | TUR | 1979 | 151.02 | 0.74 |
| Capdur | w | Old variety | FRA | 1981 | 151.80 | 0.80 |
| Cliodur | w | Modern variety | AUT | 2011 | 157.28 | 0.78 |
| CLOVIS | s | Modern variety | FRA | 2009 | 150.63 | 0.75 |
| Condur | w | Old variety | ROM | 1999 | 149.17 | 1.92 |
| Condurum | w | Old variety | ROM | 1999 | 148.84 | 1.56 |
| DAKTER | s | Modern variety | FRA | 2005 | 149.49 | 1.66 |
| Delfin | w | Modern variety | UKR | 2000 | 151.19 | 0.83 |
| Delta | w | Old variety | UKR | 1997 | 150.54 | 0.71 |
| DF329-77 | w | Old breeding line | ROM | NA | 152.54 | 0.81 |
| DF434-78 | w | Old breeding line | ROM | NA | 157.90 | 0.78 |
| DonPedro | w | Old variety | ESP | 1987 | 146.38 | 1.67 |
| DonRicardo | s | Modern variety | ESP | 2008 | 147.71 | 1.96 |
| Ducados | s | Modern variety | ESP | 2011 | 150.05 | 0.80 |
| Duramant | s | Modern variety | AUT | 2013 | 152.62 | 0.79 |
| Duramar | w | Modern variety | AUT | 2000 | 150.23 | 0.78 |
| Duramonte | s | Modern variety | ESP | 2011 | 154.66 | 0.81 |
| Durasol | w | Modern variety | DEU | 2008 | 152.50 | 0.77 |
| Durasur | s | Modern variety | ESP | 2012 | 151.30 | 0.74 |
| Duratec | s | Modern variety | ESP | 2009 | 148.62 | 0.77 |
| Durobonus | w | Modern variety | AUT | 2004 | 151.92 | 1.83 |
| Duromax | s | Modern variety | AUT | 2011 | 151.30 | 0.76 |
| Duronesse | s | Modern variety | ESP | 2013 | 150.43 | 0.78 |
| Elsadur | w | Modern variety | AUT | 2009 | 151.52 | 0.80 |
| FABULIS | s | Modern variety | FRA | 2010 | 150.50 | 1.91 |
| Floradur | w | Modern variety | AUT | 2003 | 151.66 | 0.78 |
| FURIOCAMILLO | s | Modern variety | ITA | 2012 | 145.64 | 0.92 |
| Gardemarin | w | Modern variety | UKR | 2006 | 150.41 | 1.83 |
| Gelios | w | Modern variety | RUS | 2005 | 150.64 | 0.80 |
| GKAga | w | Old variety | HUN | 1985 | 152.12 | 0.82 |
| GKBetadur | w | Old variety | HUN | 1996 | 151.22 | 0.86 |
| GKJulidur | w | Old variety | HUN | NA | 155.57 | 0.76 |
| GKMinaret | w | Old variety | HUN | 1980 | 151.05 | 0.82 |
| GKNovodur | w | Old variety | HUN | 1993 | 152.34 | 0.83 |
| GKPannondur | w | Old variety | HUN | 1985 | 150.01 | 1.72 |
| GKSelyemur | w | Modern variety | HUN | 2001 | 156.84 | 0.74 |
| GKTiszadur | w | Old variety | HUN | 1992 | 151.29 | 0.76 |
| Gordeiforme1144/a | w | Old variety | NA | NA | 152.74 | 0.80 |
| Gordeiforme1443 | w | Old variety | NA | NA | 149.79 | 0.79 |
| Gordeiforme6 | w | Modern variety | RUS | 2009 | 150.09 | 0.81 |
| Grandur | w | Old variety | AUT | 1980 | 148.64 | 0.79 |
| GUS8-bcxa-08/96 | w | Modern breeding line | USA | NA | 152.90 | 0.83 |
| Hisasano | s | Modern variety | NA | NA | 147.36 | 1.88 |
| Isildur | s | Modern variety | SVK | 2007 | 151.63 | 1.79 |
| ISPentadur | w | Modern variety | FRA | 2007 | 149.93 | 0.82 |
| Istrodur | w | Old variety | NA | NA | 150.89 | 1.92 |
| Ittu | w | NA | NA | NA | 149.99 | 1.99 |
| Jaschma | w | Old variety | NA | 1990 | 150.39 | 0.79 |
| Karur | s | Modern variety | FRA | 2002 | 150.74 | 0.76 |
| Kermen | w | Modern variety | RUS | 2006 | 150.82 | 0.73 |
| Kharkovskaya32 | w | Old variety | UKR | 1997 | 148.40 | 0.63 |
| Kiradur | w | Modern variety | AUT | 2011 | 151.84 | 0.79 |
| Kiziltan91 | w | Old variety | TUR | 1991 | 152.54 | 0.81 |
| Kontinent | w | Modern variety | UKR | 2008 | 148.62 | 0.78 |
| Koralodesskij-1 | w | Old variety | UKR | 1984 | 151.89 | 0.84 |
| Krupinka | w | Modern variety | RUS | 2005 | 151.99 | 0.75 |
| Kurant | w | Modern variety | RUS | 2004 | 149.49 | 0.77 |
| Lagoon | w | Old variety | NA | NA | 150.54 | NA |
| Leukurum21 | w | Old variety | RUS | 1996 | 150.18 | 0.81 |
| Leukurum479 | w | Old variety | UKR | NA | 151.07 | 0.75 |
| LEVANTE | s | Modern variety | ITA | 2002 | 150.94 | 2.04 |
| Liberdur | s | Modern variety | FRA | 2007 | 151.57 | 1.89 |
| Lloyd | w | Old variety | USA | 1983 | 151.79 | 0.81 |
| Logidur | w | Modern variety | AUT | 2008 | 154.28 | 0.79 |
| Luminur | s | Modern variety | FRA | 2011 | 152.77 | 0.76 |
| Lunadur | w | Modern variety | AUT | 2006 | 153.37 | 0.82 |
| Lupidur | w | Modern variety | AUT | 2009 | 154.77 | 0.80 |
| Malvadur | s | Modern variety | AUT | 2010 | 151.30 | 0.78 |
| MARCOAURELIO | s | Modern variety | ITA | 2010 | 148.34 | 1.82 |
| Martondur1 | w | Old variety | HUN | 1995 | 151.06 | 0.81 |
| Martondur3 | w | Old variety | HUN | 1999 | 154.44 | 0.77 |
| Meridionao | w | Old variety | ITA | 1999 | 146.55 | 1.70 |
| Miradoux | w | Modern variety | FRA | 2007 | 151.90 | 0.75 |
| MONASTIR | s | Modern variety | ITA | 2009 | 149.63 | 0.78 |
| Montero | s | Modern variety | NA | NA | 147.12 | 0.77 |
| Montferrier | w | Old variety | FRA | 1962 | 143.63 | 0.50 |
| MV05/08 | w | Modern breeding line | HUN | NA | 149.17 | 0.79 |
| MVGyemant | w | Modern variety | HUN | 2004 | 152.27 | 0.77 |
| MVMakaroni | w | Modern variety | HUN | 2001 | 157.64 | 0.80 |
| MVMaxidur | w | Modern variety | HUN | 2001 | 153.61 | 0.78 |
| Mvtd04-08 | w | Modern breeding line | HUN | NA | 150.48 | 0.82 |
| Mvtd07-08 | w | Modern breeding line | HUN | NA | 149.87 | 0.81 |
| Mvtd122-11 | w | Modern breeding line | HUN | NA | 156.30 | 0.77 |
| Mvtd124-11 | w | Modern breeding line | HUN | NA | 153.67 | 0.85 |
| Mvtd129-12 | w | Modern breeding line | HUN | NA | 152.85 | 1.31 |
| Mvtd13-11 | w | Modern breeding line | HUN | NA | 149.90 | 0.76 |
| Mvtd136-12 | w | Modern breeding line | HUN | NA | 150.17 | 0.76 |
| MVTD15-10 | w | Modern breeding line | HUN | NA | 156.54 | 0.80 |
| Mvtd16-11 | w | Modern breeding line | HUN | NA | 152.54 | 0.76 |
| MVTD20-12 | w | Modern breeding line | HUN | NA | 152.08 | 0.80 |
| MVTD21-11 | w | Modern breeding line | HUN | NA | 154.09 | 0.80 |
| MVTD23-11 | w | Modern breeding line | HUN | NA | 151.26 | 0.77 |
| MVTD23-12 | w | Modern breeding line | HUN | NA | 152.84 | 1.95 |
| NA | s | Modern variety | ITA | 2001 | 145.51 | 1.81 |
| NA | s | Old variety | ITA | 1998 | 147.80 | 1.08 |
| NA | s | Modern variety | ITA | 2008 | 146.25 | 1.80 |
| NA | s | Modern variety | ITA | 1996 | 145.22 | 1.86 |
| NA | s | Modern variety | ESP | 2005 | 145.13 | 0.88 |
| NA | s | Modern variety | FRA | 2010 | 149.45 | 1.83 |
| NA | s | Old variety | ITA | 1996 | 147.07 | 2.04 |
| NA | s | Modern variety | ITA | 2007 | 144.93 | 1.97 |
| NA | w | Old variety | ROM | 1984 | 149.43 | 1.32 |
| NA | w | Modern variety | AUT | 2013 | 157.42 | 0.79 |
| NEFER | s | Modern variety | FRA | 1996 | 150.28 | 1.84 |
| Neodur | s | Old variety | FRA/ITA | 1987 | 149.98 | 0.77 |
| Nobilis | s | Modern variety | FRA | 2013 | 151.95 | 1.76 |
| Novinka2 | w | Old variety | RUS | 1982 | 147.96 | 0.82 |
| Odessa65 | w | Old variety | UKR | 1984 | 152.02 | 0.75 |
| Odessa66 | w | Old variety | UKR | 1983 | 150.01 | 2.01 |
| Odmadur1 | w | Old variety | UKR | 1982 | 150.05 | 0.83 |
| Odmadur2 | w | Old variety | UKR | 1983 | 151.04 | 0.83 |
| Orjaune | s | Modern variety | DEU | 1995 | 152.98 | 0.73 |
| ORLU | s | Modern variety | FRA | 2002 | 149.98 | 0.77 |
| Pandur | w | Old variety | AUT | 1996 | 149.89 | 1.93 |
| Parus | w | Old variety | UKR | 1983 | 151.28 | 0.77 |
| Perlynaodeska | w | Modern variety | UKR | 2002 | 151.67 | 0.77 |
| PESCADOU | s | Modern variety | FRA | 2002 | 150.65 | 0.80 |
| Plussur | s | Modern variety | FRA | 2011 | 152.83 | 0.75 |
| PR22D89 | s | Modern breeding line | NA | NA | 146.53 | 1.78 |
| PRECO | s | Modern variety | ITA | 2007 | 147.16 | 1.02 |
| Primadur | w | Old variety | FRA | 1984 | 149.94 | 0.83 |
| Prowidur | w | Modern variety | AUT | 2000 | 156.59 | 0.77 |
| Quadrato | s | Old variety | ITA | 1999 | 149.43 | 1.82 |
| Qualidou | s | Modern variety | FRA | 2011 | 151.26 | 0.80 |
| RGT Izalmur | s | Modern variety | FRA | 2015 | 154.20 | 0.72 |
| RGT Nomur | s | Modern variety | FRA | 2015 | 154.06 | 0.77 |
| S-10005-103-303/5-402/2/1 | s | Modern breeding line | NA | NA | 152.82 | 0.79 |
| S-10007-104-304/2-403/2/3 | s | Modern breeding line | NA | NA | 148.31 | 0.75 |
| S-10019-R211-R/411/1-523/3/1 | s | Modern breeding line | NA | NA | 147.94 | 0.76 |
| S-10022-109-309/6-405/1/1 | s | Modern breeding line | NA | NA | 151.10 | 0.76 |
| S-10041-124-324/1-406/3/1 | s | Modern breeding line | NA | NA | 150.70 | 1.15 |
| S-10043-125-325/4-408/4/1 | s | Modern breeding line | NA | NA | 153.03 | 0.73 |
| S-10046-128-328/1-412/1/1 | s | Modern breeding line | NA | NA | 153.92 | 0.80 |
| S-10046-128-328/3-413/6/3 | s | Modern breeding line | NA | NA | 154.82 | 0.76 |
| S-10050-132-332/1-416/1/3 | s | Modern breeding line | NA | NA | 153.02 | 0.76 |
| S-10053-135-335/7-419/1/3 | s | Modern breeding line | NA | NA | 152.02 | 0.75 |
| S-10053-135-335/9-421/5/3 | s | Modern breeding line | NA | NA | 152.98 | 0.77 |
| S-10066-140-340/10(B)-427/5/3 | s | Modern breeding line | NA | NA | 152.49 | 0.77 |
| S-10070-141-341/5-432/1/2 | s | Modern breeding line | NA | NA | 152.91 | 0.72 |
| S-10073-142-342/2-437/4/3 | s | Modern breeding line | NA | NA | 154.06 | 0.76 |
| S-10075-R227-R/427/2-528/5/2 | s | Modern breeding line | NA | NA | 154.01 | 0.76 |
| S-10081-145-345/3-445/1/3 | s | Modern breeding line | NA | NA | 150.96 | 0.76 |
| S-10081-145-345/4-446/6/1 | s | Modern breeding line | NA | NA | 152.37 | 0.78 |
| S-10085-147-347/2-447/4/3 | s | Modern breeding line | NA | NA | 151.74 | 0.76 |
| S-10093-152-352/2-449/1/1 | s | Modern breeding line | NA | NA | 153.18 | 0.75 |
| S-10093-152-352/4-451/4/3 | s | Modern breeding line | NA | NA | 155.60 | 0.76 |
| S-10093-152-352/7-454/1/1 | s | Modern breeding line | NA | NA | 150.31 | 0.74 |
| S-10094-153-353/2-456/5/1 | s | Modern breeding line | NA | NA | 150.74 | 0.75 |
| S-10096-R237-R/437/1-532/2/2 | s | Modern breeding line | NA | NA | 152.23 | 0.75 |
| S-10096-R237-R/437/2-533/3/1 | s | Modern breeding line | NA | NA | 152.01 | 0.72 |
| S-10099-156-356/2-466/2/3 | s | Modern breeding line | NA | NA | 149.88 | 0.75 |
| S-10100-157-357/1-468/3/3 | s | Modern breeding line | NA | NA | 154.43 | 0.76 |
| S-10104-R241-R/441/1-536/2/2 | s | Modern breeding line | NA | NA | 153.07 | 0.76 |
| S-10106-158-358/1-471/4/3 | s | Modern breeding line | NA | NA | 150.65 | 0.80 |
| S-10108-R244-R/444/2-539/1/1 | s | Modern breeding line | NA | NA | 151.20 | 0.75 |
| S-10113-162-362/6-477/1/1 | s | Modern breeding line | NA | NA | 152.44 | 0.77 |
| S-10113-162-362/9-479/3/3 | s | Modern breeding line | NA | NA | 153.29 | 0.75 |
| S-10118-165-365/3-484/2/3 | s | Modern breeding line | NA | NA | 151.65 | 0.75 |
| S-10118-165-365/5-486/4/3 | s | Modern breeding line | NA | NA | 151.74 | 0.70 |
| S-10121-167-367/10-493/1/1 | s | Modern breeding line | NA | NA | 152.87 | 0.77 |
| S-10121-167-367/2-495/1/3 | s | Modern breeding line | NA | NA | 151.44 | 0.75 |
| S-10121-167-367/5-497/5/1 | s | Modern breeding line | NA | NA | 152.52 | 0.77 |
| S-10121-167-367/9-501/1/3 | s | Modern breeding line | NA | NA | 150.65 | 0.76 |
| S-63/05-382/1-382/1-508/3/3 | s | Modern breeding line | NA | NA | 150.50 | 0.73 |
| S-63/05-384/1-384/1-511/3/1 | s | Modern breeding line | NA | NA | 149.42 | 0.69 |
| SARAGOLLA | s | Modern variety | ITA | 2004 | 146.62 | 1.80 |
| Sculptur | s | Modern variety | FRA | 2007 | 147.97 | 1.80 |
| Selcuklu | w | Old variety | TUR | 1997 | 150.72 | 0.77 |
| Simeto | s | Old variety | ITA | 1988 | 141.99 | 1.81 |
| Snowglenn | w | Modern variety | USA | 2008 | 150.63 | 1.98 |
| Soldur | w | Old variety | SVK | 1989 | 155.14 | 0.77 |
| SVEVO | s | Old variety | ITA | 1996 | 146.29 | 1.94 |
| SZD1977 | w | Modern breeding line | AUT | NA | 150.89 | 0.78 |
| SZD2108 | w | Modern breeding line | AUT | NA | 154.44 | 0.79 |
| SZD2171 | w | Modern breeding line | AUT | NA | 155.68 | 0.77 |
| SZD2371D | w | Modern breeding line | AUT | NA | 157.58 | 0.81 |
| SZD2384BD | s | Modern breeding line | AUT | NA | 152.25 | 0.76 |
| SZD2384BH | s | Modern breeding line | AUT | NA | 153.10 | 0.77 |
| SZD2399A | w | Modern breeding line | AUT | NA | 153.83 | 0.82 |
| SZD2399B | w | Modern breeding line | NA | NA | 153.91 | 0.78 |
| SZD2434A | w | Modern breeding line | AUT | NA | 152.15 | 0.86 |
| SZD2522 | s | Modern breeding line | NA | NA | 153.40 | 0.77 |
| SZD2774B | w | Modern breeding line | AUT | NA | 152.39 | 0.83 |
| SZD2817A | w | Modern breeding line | AUT | NA | 155.01 | 0.78 |
| SZD2929 | w | Modern breeding line | AUT | NA | 151.73 | 0.84 |
| SZD2929_2760Bl8 | w | Modern breeding line | AUT | NA | 153.82 | 0.77 |
| SZD2937 | w | Modern breeding line | AUT | NA | 148.84 | 0.79 |
| SZD3105 | s | Modern breeding line | NA | NA | 149.66 | 0.86 |
| Tablur | s | Modern variety | FRA | 2010 | 149.93 | 0.77 |
| TD4-MARTIN | s | Modern variety | NA | NA | 147.53 | 1.97 |
| TRAPEZIO | s | Modern variety | GRC | 2006 | 150.76 | 0.78 |
| Tredur | s | Modern variety | ESP | 2012 | 146.88 | 0.76 |
| Troubadur | w | Old variety | AUT | NA | 150.27 | 0.88 |
| TT04DD79IN27 | w | Modern breeding line | FRA | NA | 149.56 | 0.77 |
| VA05WD-12 | w | Modern breeding line | USA | NA | 150.94 | 0.81 |
| VA05WD-31 | w | Modern breeding line | USA | NA | 151.41 | 0.80 |
| VA05WD-39 | w | Modern breeding line | USA | NA | 151.49 | 0.76 |
| Vitrosol | s | Modern variety | ESP | 2008 | 149.44 | 0.73 |
| W-09002-101-201-301/14-404/1/1 | w | Modern breeding line | NA | NA | 154.24 | 0.76 |
| W-09002-101-201-301/4-407/4/3 | w | Modern breeding line | NA | NA | 154.55 | 0.74 |
| W-09004-102-202-302/8-420/1/3 | w | Modern breeding line | NA | NA | 154.65 | 0.71 |
| W-09004-102-202-302/9-421/3/2 | w | Modern breeding line | NA | NA | 153.42 | 0.75 |
| W-09008-104-204-304/9-435/1/3 | w | Modern breeding line | NA | NA | 155.66 | 0.71 |
| W-09014-107-207-307/10-447/1/3 | w | Modern breeding line | NA | NA | 157.85 | 0.78 |
| W-09014-107-207-307/1-446/3/1 | w | Modern breeding line | NA | NA | 156.08 | 0.74 |
| W-09014-107-207-307/6-451/1/3 | w | Modern breeding line | NA | NA | 155.71 | 0.79 |
| W-09016-108-208-308/9-460/2/3 | w | Modern breeding line | NA | NA | 158.84 | 0.77 |
| W-09018-109-209-309/5-464/2/1 | w | Modern breeding line | NA | NA | 153.57 | 0.76 |
| W-09018-109-209-309/7-466/1/3 | w | Modern breeding line | NA | NA | 156.44 | 0.74 |
| W-09020-110-210-310/1-469/1/3 | w | Modern breeding line | NA | NA | 152.34 | 0.77 |
| W-09020-110-210-310/4-471/2/3 | w | Modern breeding line | NA | NA | 154.51 | 0.72 |
| W-09020-110-210-310/8-474/1/3 | w | Modern breeding line | NA | NA | 154.91 | 0.66 |
| W-09020-110-210-310/9-475/5/3 | w | Modern breeding line | NA | NA | 154.78 | 0.70 |
| W-09026-113-212-312/10-487/4/1 | w | Modern breeding line | NA | NA | 156.65 | 0.69 |
| W-09026-113-212-312/12-489/1/3 | w | Modern breeding line | NA | NA | 155.86 | 0.75 |
| W-09028-114-213-313/2-503/1/1 | w | Modern breeding line | NA | NA | 153.90 | 0.75 |
| W-09028-114-213-313/3-504/6/3 | w | Modern breeding line | NA | NA | 153.97 | 0.72 |
| W-09028-114-213-313/4-505/3/1 | w | Modern breeding line | NA | NA | 157.09 | 0.73 |
| W-09028-114-213-313/8-508/1/1 | w | Modern breeding line | NA | NA | 153.65 | 0.70 |
| W-09028-114-213-313/9-509/2/3 | w | Modern breeding line | NA | NA | 154.10 | 0.75 |
| W-09030-115-214-314/2-512/3/3 | w | Modern breeding line | NA | NA | 153.70 | 0.71 |
| W-09030-115-214-314/3-513/1/3 | w | Modern breeding line | NA | NA | 156.05 | 0.76 |
| W-09030-115-214-314/5-515/3/3 | w | Modern breeding line | NA | NA | 154.91 | 0.75 |
| W-09030-115-214-314/7-517/1/3 | w | Modern breeding line | NA | NA | 155.68 | 0.71 |
| W-09030-115-214-314/9-519/3/3 | w | Modern breeding line | NA | NA | 155.71 | 0.76 |
| W-09031-116-215-315/2-522/2/3 | w | Modern breeding line | NA | NA | 153.62 | 0.74 |
| W-09033-117-216-316/8-532/2/3 | w | Modern breeding line | NA | NA | 152.53 | 0.68 |
| W-09050-130-228-328/2-563/1/3 | w | Modern breeding line | NA | NA | 154.84 | 0.78 |
| W-09050-130-228-328/3-564/1/1 | w | Modern breeding line | NA | NA | 156.04 | 0.75 |
| W-09058-138-234-334/4-569/1/3 | w | Modern breeding line | NA | NA | 154.16 | 0.74 |
| Windur | w | Old variety | DEU | 1984 | 153.95 | 0.81 |
| Wintergold | w | Modern variety | DEU | 2011 | 153.95 | 0.77 |
| XVAD99068-14 | w | Modern breeding line | USA | NA | 152.28 | 1.11 |
| XVAD99069-18 | w | Modern breeding line | USA | NA | 149.67 | 1.88 |
| XVAD99142-10 | w | Modern breeding line | USA | NA | 151.13 | 0.82 |
| Yilmaz98 | w | Old variety | TUR | 1998 | 149.84 | 0.74 |
| Yukon | w | Modern variety | DEU | 2005 | 154.40 | 0.75 |
| Zaperozhskaja | w | Old variety | NA | 1990 | 151.49 | 0.81 |
| ZoloteRuno | w | Modern variety | UKR | 2004 | 150.78 | 0.76 |

^1^ Genotype classified as spring (s) or winter (w) type

**Additional file 1: Table S2.** Summary statistics for heading time.

|  | Heading time (days)^1^ |
| --- | --- |
| Min. | 141.99 |
| Mean | 152.09 |
| Max. | 161.96 |
| $\sigma_{G}^{2}$ | 7.52** |
| $\sigma_{G\times E}^{2}$ | 0.83** |
| $\sigma_{e}^{2}$ | 2.24 |
| *h^2^* | 0.88 |

^1^ Reported by Miedaner et al. [17]

**Additional file 1: Table S3.** Correlations between the significantly associated markers on chromosome 2B and *Ppd-B1* copy number variation (ratio *Ppd-B1* / *TaCO2*).

| Marker | Chr. | Pos. (cM) | Pos. (Mbp)^1^ | *r_Ppd-B1_* |
| --- | --- | --- | --- | --- |
| D3935165 | 2B | 36.35 | 53,704,532 | 0.90 |
| S1713466 | 2B | 36.35 | 53,972,352 | 0.87 |
| S2279856 | 2B | 37.15 | 56,191,088 | 0.91 |
| D1099896 | 2B | 39.51 | 53,406,376 | 0.65 |
| S1106958 | 2B | 40.74 | 53,701,140 | 0.90 |
| D12735838 | 2B | 40.74 | 53,067,983 | 0.89 |
| S3021610 | 2B | 40.74 | 53,972,355 | 0.90 |
| D4004228 | 2B | 40.74 | 56,011,661 | 0.91 |
| S1353553 | 2B | 40.74 | 54,098,441 | 0.88 |
| D6040039 | 2B | 40.74 | 53,972,355 | 0.88 |
| S986135 | 2B | 40.99 | 54,516,891 | 0.92 |
| S1124640 | 2B | 41.86 | 54,468,610 | 0.88 |
| S1128199 | 2B | 61.42 | - | 0.46 |

^1^ Physical position on the durum wheat reference genome Maccaferri et al. [35]


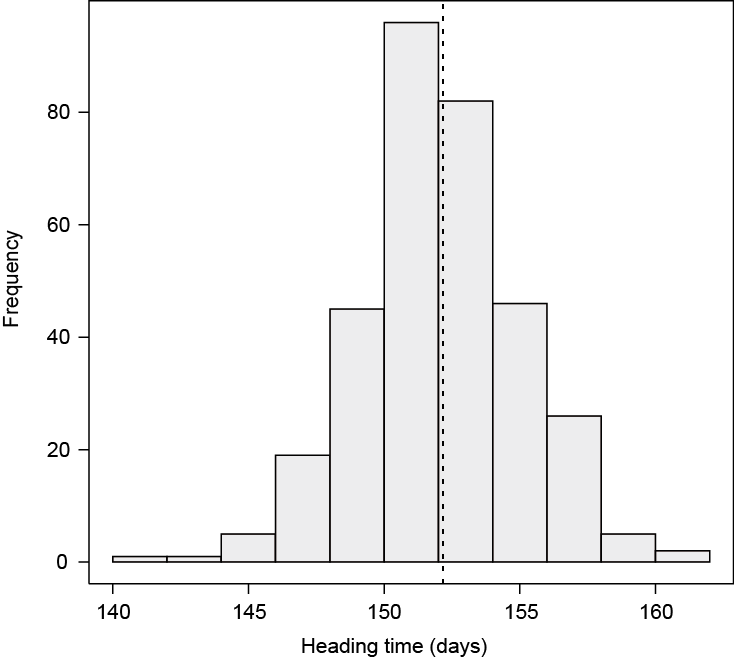


**Additional file 1: Figure S1.** Histogram of the heading time BLUEs.


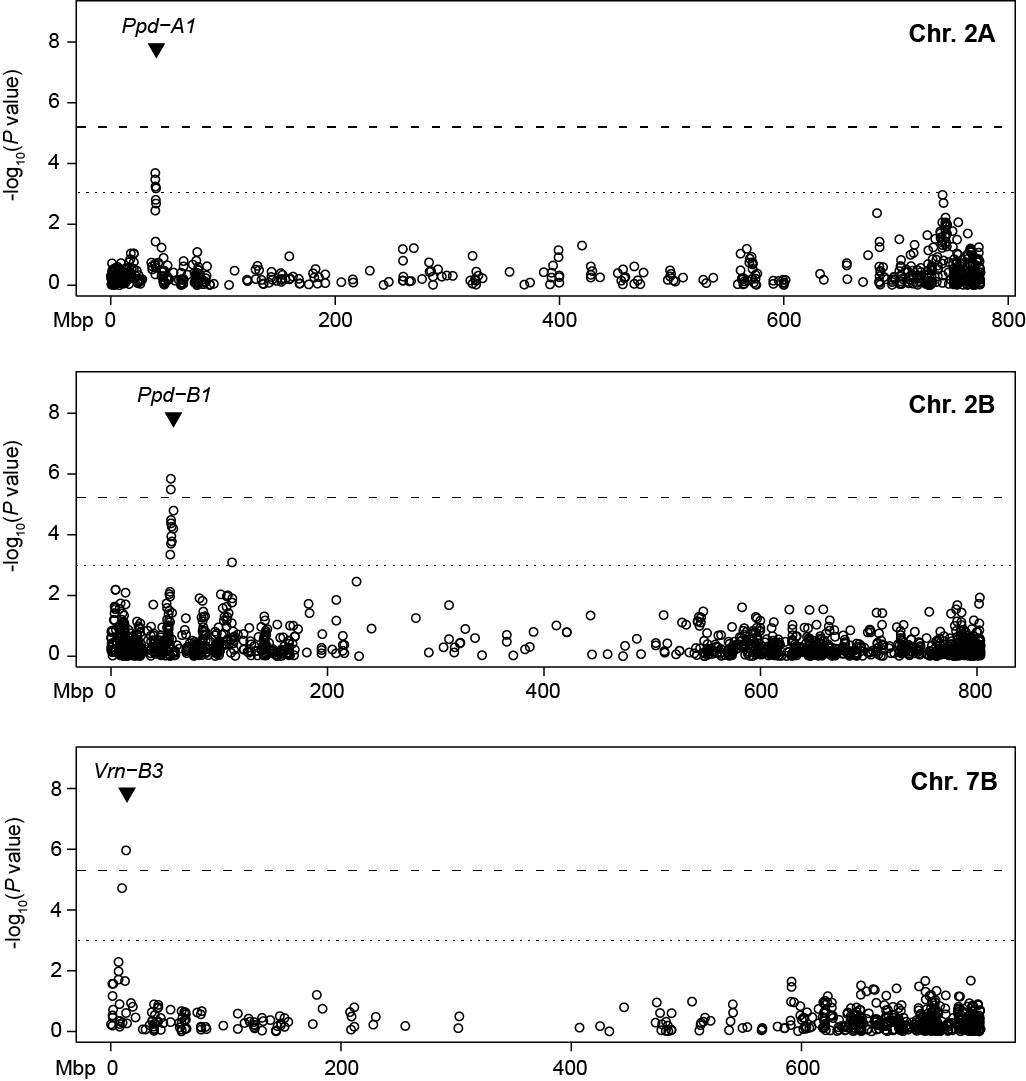


**Additional file 1: Figure S2.** Results from the genome-wide scan for marker-trait association for heading time for chromosomes 2A, 2B and 7B, with the markers plotted according to their physical position in the wild emmer reference genome [36]. The positions of the phenology loci *Ppd-A1*, *Ppd-B1* and *Vrn-B3* are indicated by arrowheads. The dashed line indicates the significance threshold (Bonferroni-corrected *P* < 0.01) and the dotted line the exploratory threshold (*P* < 0.001).


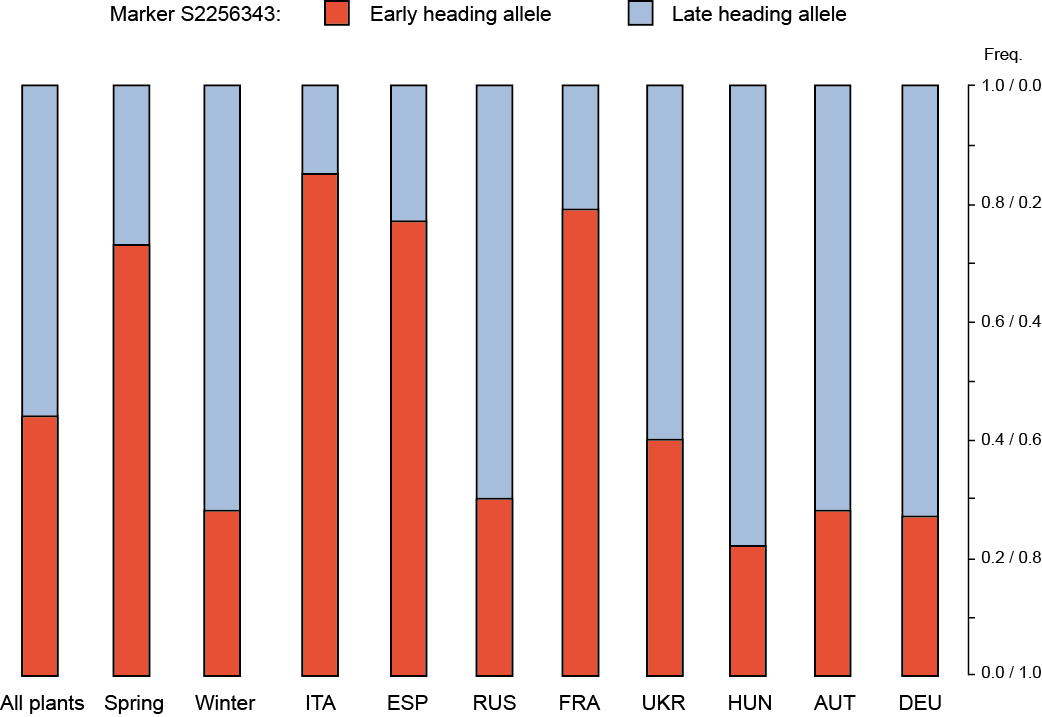


**Additional file 1: Figure S3.** Allele frequency of marker S2256343, as a proxy for *Ppd-A1*, dependent on the cultivars’ country of origin. ITA, Italy; ESP, Spain; RUS, Russian Federation; FRA, France; UKR, Ukraine; HUN, Hungary; AUT, Austria; DEU, Germany.
